# Colistin Resistance in Critically Ill Adults: A Systematic Review of Epidemiological, Clinical, Laboratory, and Molecular Evidence

**DOI:** 10.3390/microorganisms14091908

**Published:** 2026-08-28

**Authors:** Eugen Mihail Arnautu, Elena Leocadia Plesea, Ovidiu Mircea Zlatian, Georgiana Cristiana Camen, Radu Razvan Mititelu, Andrei Osman, Maria-Loredana Tieranu, Andreea Loredana Golli, Alice Elena Ghenea

**Affiliations:** 1 Doctoral School, University of Medicine and Pharmacy of Craiova, 200349 Craiova, Romania; eugen.arnautu@umfcv.ro; 2 Department of Microbiology, University of Medicine and Pharmacy of Craiova, 200349 Craiova, Romania; ovidiu.zlatian@umfcv.ro (O.M.Z.); radu.mititelu@umfcv.ro (R.R.M.); alice.ghenea@umfcv.ro (A.E.G.); 3 Radiology Department, University of Medicine and Pharmacy of Craiova, 200638 Craiova, Romania; 4 Department of Anatomy and Embriology, University of Medicine and Pharmacy of Craiova, 200349 Craiova, Romania; andrei.osman@umfcv.ro; 5 Department of Obstetrics and Gynecology, Emergency County Hospital Craiova, 200642 Craiova, Romania; loredanapospai@yahoo.com; 6 Department of Public Health and Management, University of Medicine and Pharmacy of Craiova, 200349 Craiova, Romania; andreea.golli@umfcv.ro

**Keywords:** colistin resistance, intensive care unit, antimicrobial resistance, broth microdilution, heteroresistance, *mcr*, *mgrB*, Gram-negative bacteria

## Abstract

Colistin resistance is an increasing concern in critically ill adults, but reported rates, clinical consequences, laboratory detection, and resistance mechanisms vary widely between studies. This systematic review evaluated epidemiological, clinical, laboratory, and molecular evidence on colistin resistance in adult intensive care populations. MEDLINE/PubMed, the Cochrane Library, Scopus, and Web of Science Core Collection were searched, and the findings were synthesised descriptively because of substantial differences between studies. A total of 120 studies were included. In studies using BMD in broader ICU populations, colistin non-susceptibility ranged from approximately 0.3–57.4% at the isolate level, while patient-level carriage/acquisition estimates ranged from approximately 1.2% to 9.4%. Clinical studies often reported poorer outcomes in patients with colistin-resistant infections. However, the strength and direction of these associations were inconsistent, and most comparative analyses had substantial risk of bias. Laboratory methods influenced resistance detection, while heteroresistance required specific testing approaches. Molecular studies identified several resistance pathways, including *mcr* genes, *mgrB* disruption, regulatory alterations, clonal spread, and within-host evolution. Overall, colistin resistance in critical care is highly heterogeneous and should be interpreted according to the clinical, microbiological, and epidemiological context.

## 1. Introduction

Antimicrobial resistance is a major threat to modern healthcare. Drug-resistant Gram-negative pathogens are particularly important in intensive care units (ICUs), where severe illness, invasive procedures, antimicrobial exposure, and cross-transmission converge [1]. The 2024 WHO priority list classifies carbapenem-resistant *Acinetobacter baumannii* and *Enterobacterales* as critical-priority pathogens and carbapenem-resistant *Pseudomonas aeruginosa* as high priority [2].

Colistin (polymyxin E) remains relevant for selected multidrug- and carbapenem-resistant Gram-negative infections, especially where newer agents are unavailable. However, its use is constrained by a narrow therapeutic window, complex pharmacokinetics, and nephrotoxicity [3,4]. Current guidance increasingly favours newer active agents when microbiologically appropriate [5]; loss of colistin activity therefore marks a particularly restricted therapeutic setting.

Resistance may arise through chromosomal pathways affecting lipid A, including *mgrB*, PmrAB/PhoPQ, and lipid-A biosynthesis, or through transferable *mcr* genes [6]. Genotype does not invariably predict phenotype. Laboratory ascertainment is also difficult: broth microdilution (BMD) is the reference framework, whereas diffusion- and gradient-based methods may misclassify isolates [7]. Heteroresistance can further require dedicated detection of resistant subpopulations.

Reported ICU burden consequently depends on species, geography, patient- versus isolate-level denominators, resistance enrichment, and susceptibility-testing methods. Clinical studies additionally vary in infection syndrome, baseline severity, treatment, follow-up, and confounding. A previous meta-analysis demonstrated substantial regional variation [8], but prevalence alone does not capture the clinical, methodological, and molecular dimensions of the problem.

This systematic review therefore evaluated four domains in critically ill adults: epidemiological prevalence and burden; clinical outcomes and factors associated with resistance or acquisition; susceptibility-testing methodology and heteroresistance; and molecular determinants and epidemiology. The primary outcome was phenotypic colistin non-susceptibility, with patient- and isolate-level denominators kept separate; secondary outcomes included acquisition, mortality and other clinical outcomes, AST performance, heteroresistance, and resistance mechanisms.

## 2. Materials and Methods

### 2.1. Review Design and Reporting

The review was conducted and reported according to PRISMA 2020 [9]. Given substantial clinical and methodological heterogeneity, evidence was organised by outcome domain and synthesised narratively rather than statistically pooled.

### 2.2. Protocol and Registration

The review was not prospectively registered. Eligibility criteria, search strategy, and the analytical framework were defined before study selection.

### 2.3. Eligibility Criteria

Eligibility was defined using a Population–Concept–Context framework suitable for the review’s multiple evidence domains.

#### 2.3.1. Population, Concept, and Context

Adults aged ≥18 years admitted to an ICU or explicitly described as critically ill were eligible. Mixed ICU/non-ICU or adult/paediatric studies were included only when eligible adult ICU data were separately extractable. A narrowly defined exception allowed a predominantly adult ICU dataset with a small identifiable paediatric minority solely for AST-method comparison, not for adult prevalence or clinical outcomes. Eligible evidence concerned phenotypic colistin resistance/non-susceptibility, acquisition or emergence, and ICU-linked molecular determinants in clinically relevant Gram-negative organisms. Molecular findings were kept separate from phenotype unless established in the same isolates.

#### 2.3.2. Outcomes and Analytical Frameworks

The primary outcome was the patient- or isolate-level proportion of phenotypic colistin non-susceptibility. Secondary outcomes were acquisition/emergence, mortality and other clinical outcomes, associated factors, performance of non-reference AST methods, heteroresistance, and molecular determinants. Comparative clinical studies could use colistin resistance as the exposure and susceptible patients/isolates as the comparator. AST studies evaluated an index method against an appropriate reference standard.

#### 2.3.3. Eligible Study Designs and Exclusion Criteria

Eligible designs included surveillance, cohort, case–control, cross-sectional/prevalence, diagnostic/AST, outbreak, randomised or cluster-randomised studies reporting relevant resistance outcomes, and molecular epidemiology studies. Studies were excluded for:

• Non-critical-care populations without separable ICU data;
• Paediatric or inseparable mixed-age populations (apart from the AST exception above);
• Absence of a colistin-specific resistance outcome;
• Animal or purely experimental work without eligible ICU isolates;
• Pharmacokinetic, efficacy, safety, or toxicity studies without a resistance outcome;
• Non-primary publications;
• Environmental/veterinary/food-chain isolates without an ICU link;
• Conference abstracts lacking sufficient information.

Case reports or series with <10 patients/isolates were excluded except reports of unique ICU-linked molecular mechanisms, which contributed only to mechanistic synthesis. Overlapping publications were linked to avoid double counting, and no language restrictions were applied.

### 2.4. Information Sources and Search Strategy

MEDLINE/PubMed, the Cochrane Library, Scopus, and Web of Science Core Collection were searched from inception through 3 August 2026 without date or language restrictions. Searches combined controlled vocabulary and free text for colistin/polymyxin E, intensive care/critical illness, and resistance/non-susceptibility, including heteroresistance and *mcr*, *mgrB*, *phoPQ*, and *pmrAB*. Organism terms were not used as a separate search facet.

The final searches yielded 1915 records: 881 from MEDLINE/PubMed, 28 from Cochrane, 492 from Scopus, and 514 from Web of Science. Complete executed strategies are provided in Supplementary Table S1.

### 2.5. Study Selection

Records were deduplicated in Zotero v10 and screened in Rayyan [10] (http://rayyan.qcri.org, accessed on 3 August 2026). Two reviewers (E.M.A. and E.L.P.) independently screened titles/abstracts and assessed potentially eligible full texts; disagreements were resolved by a third reviewer (G.C.C.), and full-text exclusion reasons were recorded.

### 2.6. Data Extraction

A structured, outcome-oriented form captured study setting and design, population and sample size, organisms/specimens, colonisation or infection status, isolate selection, AST method and breakpoint, and applicable evidence domains.

Domain-specific extraction retained:

• Patient- and isolate-level numerators/denominators;
• Clinical exposure/comparator definitions, outcomes, follow-up, and crude/adjusted estimates;
• AST index/reference methods, agreement, and error data;
• Molecular methods, determinants, clonal context, and paired phenotype.

All extractable results relevant to the prespecified outcome domains were considered for data extraction and synthesis.

Genotype–phenotype concordance was assessed only in the same isolates.

Data extraction and curation were performed using a structured, outcome-oriented form by A.O. and R.R.M., with subsequent validation by A.E.G.

### 2.7. Organisation of the Evidence

The evidence was organised into four non-exclusive analytical domains: epidemiological burden; clinical outcomes/acquisition/associated factors; AST methodology/heteroresistance; and molecular determinants/epidemiology. A study could contribute to more than one domain, but the same population or isolate set was counted only once per outcome.

### 2.8. Classification of Colistin Susceptibility-Testing Methods

AST methods were classified as reference BMD, adequately documented BMD, non-reference methods (including automated, gradient-diffusion, agar-dilution, or rapid approaches), or unreliable/insufficiently reported methods (including disc diffusion or unclear procedures). For prevalence interpretation, reference or adequately documented BMD was prioritised [4,7]. The review uses “colistin non-susceptibility” for prevalence estimates, “colistin-resistant” when reproducing study classifications, and “colistin resistance” for the general phenomenon.

### 2.9. Risk of Bias and Methodological Appraisal

Two reviewers (L.M.T. and E.L.P.) performed design- and question-specific appraisal, with disagreements resolved by A.E.G. A publication could receive more than one instrument when it contributed methodologically distinct components. Judgements were interpreted within each instrument and were not converted into a common numerical score.

#### 2.9.1. Prevalence Studies—JBI

Prevalence studies were appraised with the JBI prevalence checklist [11], emphasising the representativeness of the denominator and validity/consistency of colistin susceptibility ascertainment. Review-level Low/Moderate/High categories were prespecified operational interpretations rather than official JBI scores.

#### 2.9.2. Exposure–Outcome Studies—ROBINS-E

Exposure–outcome effects were assessed result-specifically with ROBINS-E (24 March 2024 version) [12], considering confounding, exposure measurement, participant selection, post-exposure interventions, missing data, outcome measurement, and selection of the reported result. Overall judgements were low risk, some concerns, high risk, or very high risk.

#### 2.9.3. Diagnostic and Susceptibility-Testing Studies—QUADAS-3

Diagnostic/AST comparisons were assessed with QUADAS-3 v1.2 [13]. BMD was the preferred reference for conventional colistin susceptibility and population analysis profiling with MIC confirmation for heteroresistance. Formal accuracy estimates were used only when an appropriate reference standard and complete data supported valid reconstruction; otherwise, agreement, error rates, or diagnostic yield were reported descriptively.

#### 2.9.4. Other Observational and Mixed-Design Evidence—MMAT 2018

Other eligible observational or mixed-design components were appraised with MMAT 2018 [14]. MMAT criteria were interpreted individually without an overall percentage score. Complete JBI, MMAT, ROBINS-E, and QUADAS-3 study-level assessments are provided in Supplementary Table S2.

### 2.10. Data Synthesis

All four domains were synthesised narratively. Effect measures were retained in their study-reported form. Epidemiological outcomes were expressed as proportions, clinical associations as reported crude or adjusted effect estimates with confidence intervals, and AST comparisons as agreement, error, or diagnostic accuracy measures when validly supported. No missing summary statistics were imputed, and effect measures were not converted across scales. Derived measures were calculated only when they could be validly reconstructed from the reported data. Study-level findings were tabulated by evidence domain in the main text and supplementary tables, while risk-of-bias assessments were summarised graphically at criterion- or domain-level, as appropriate to each appraisal tool. Epidemiological estimates retained study-specific numerators, denominators, and proportions, kept patient- and isolate-level outcomes separate, prioritised BMD, and distinguished broader ICU populations from resistance-enriched, outbreak, or otherwise selected denominators. No overall prevalence was calculated.

The clinical results retained outcome timing, crude/adjusted effects, confidence intervals, baseline severity, treatment timing, and confounder adjustment without treating different mortality horizons or effect measures as interchangeable. AST studies were synthesised by index/reference method, with formal accuracy measures reported only when valid; heteroresistance was analysed separately. Molecular frequencies from selected sequencing, resistant, or outbreak collections were not interpreted as population prevalence, and determinants were only linked to phenotype in the same isolates. No formal sensitivity analyses were performed because the review used a narrative, domain-stratified synthesis rather than quantitative pooling. No formal assessment of the certainty of the body of evidence was performed.

## 3. Results

### 3.1. Study Selection Result

**Figure 1:**
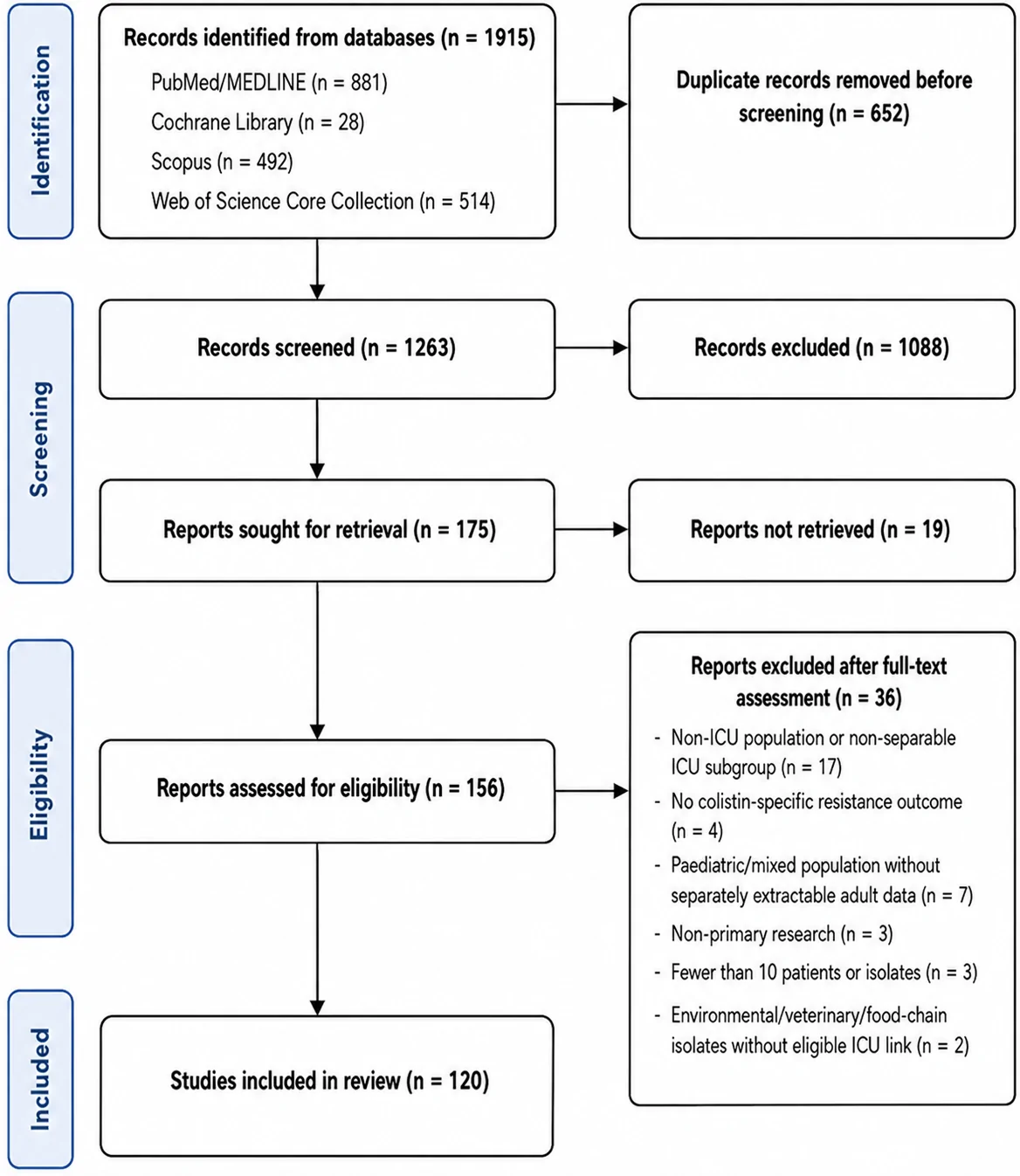
PRISMA 2020 flow diagram of study identification, screening, eligibility assessment, and inclusion.

The searches identified 1915 records; 652 duplicates were removed and 1263 unique records screened. Of 175 reports sought for retrieval, 19 were unavailable. After the assessment of 156 full texts, 36 were excluded and 120 studies/reports were included (Figure 1). Full-text exclusions were mainly non-ICU/non-separable populations (n = 17), mixed or paediatric populations without extractable adult data (n = 7), absent colistin-specific outcomes (n = 4), non-primary research (n = 3), samples < 10 (n = 3), and environmental/veterinary/food-chain isolates without an ICU link (n = 2).

### 3.2. Characteristics of Included Studies

The 120 studies spanned 2007–2026 and multiple designs and regions (Table 1). Thirty-seven contributed prevalence/surveillance evidence, 49 clinical outcomes/acquisition/associated factors, 10 AST methodology/heteroresistance, and 54 molecular/mechanistic evidence. Because domains overlapped, these represented 150 study-domain contributions: 95 studies contributed to one domain, 20 to two, and five to three. Most publications were from Türkiye (26), India (13), Iran (10), Greece (nine), and the Netherlands (eight); 75/120 (62.5%) were published in the period 2020–2026. Molecular/isolate-series epidemiology (38 studies) and prevalence/surveillance designs (22) were the most frequent named categories. *Klebsiella pneumoniae* (60 studies) and *Acinetobacter baumannii* (45) were the most frequently represented organisms, followed by *P. aeruginosa* (19) and *Escherichia coli* (14). AST reporting was variable: a BMD or BMD-associated platform was reported in 50 studies, automated systems in 34, and diffusion-based methods in 29, and the AST method was unreported or insufficiently specified in 54. AST-method categories are not mutually exclusive; a single study may report more than one testing method.

### 3.3. Risk of Bias and Methodological Quality

Appraisal was assigned by analytical contribution: 37 JBI prevalence assessments, 84 MMAT assessments, 11 result-specific ROBINS-E assessments, and four QUADAS-3 assessments. Counts exceed 120 because distinct components of some studies were appraised separately.

#### 3.3.1. JBI Prevalence Appraisal

**Figure 2:**
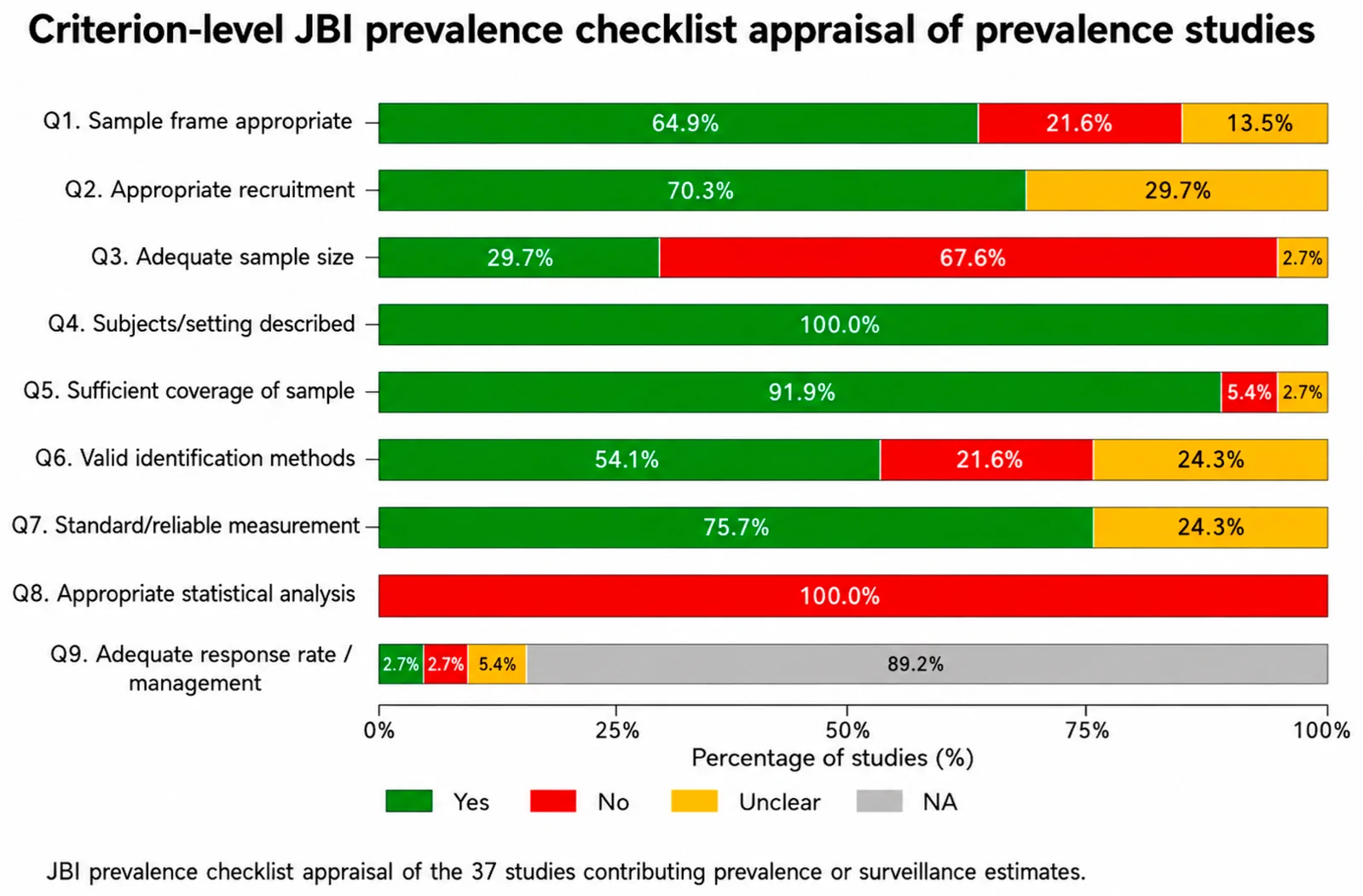
Criterion-level JBI prevalence appraisal for the 37 studies. Full assessments are presented in Supplementary Table S2. Note: None of the prevalence studies fully met JBI item 8 (appropriate statistical analysis), as no study reported confidence intervals or other measures of precision.

Among 37 JBI prevalence assessments, five (13.5%) had low, 18 (48.6%) moderate, and 14 (37.8%) high methodological concern (Figure 2). High concern most often reflected resistance-enriched/non-representative sampling or uncertain AST validity. All studies lacked confidence intervals or equivalent precision measures for prevalence, the key component of JBI item 8 (appropriate statistical analysis). At the criterion level, sampling-frame appropriateness and valid resistance ascertainment were recurring limitations, whereas description of the study setting was generally adequate. Moderate concern often reflected incomplete information on sampling, denominator completeness, or precision rather than an unequivocally invalid design.

#### 3.3.2. ROBINS-E Appraisal of Exposure–Outcome Associations

**Figure 3:**
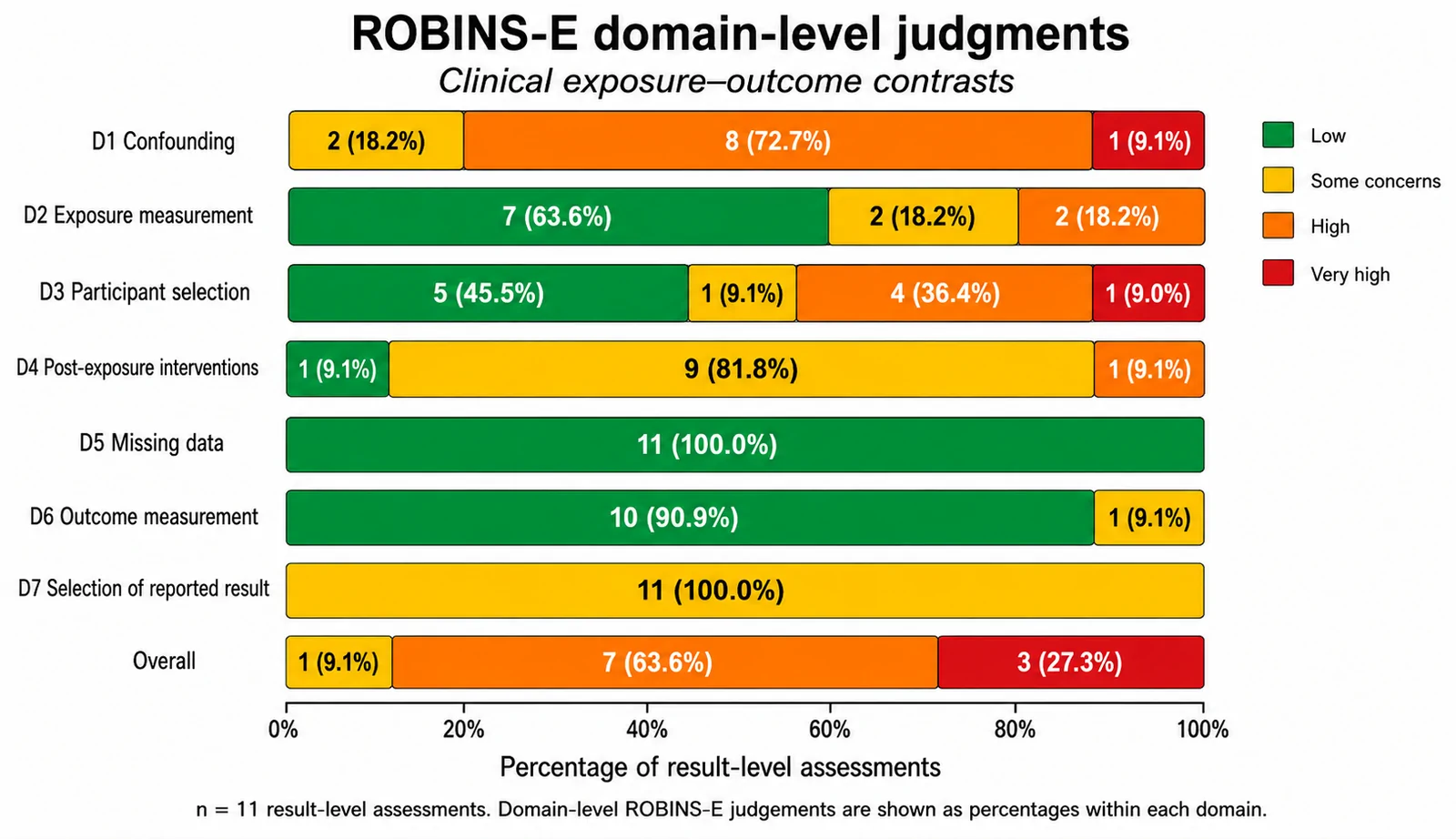
ROBINS-E domain-level judgements for the 11 result-specific exposure–outcome assessments. Full assessments are provided in Supplementary Table S2.

For the 11 ROBINS-E exposure–outcome results, three were at very high risk of bias, seven were at high risk, one had some concerns, and none were low risk (Figure 3). Residual confounding and selection/temporal alignment problems predominated, so these findings were interpreted as observational associations rather than unbiased causal effects. Several studies restricted analysis to patients who survived or remained in the ICU long enough to satisfy a study definition, excluded early deaths, or did not align resistance ascertainment with time zero. Post-exposure treatment and source-control variables also complicated causal interpretation because they may act as mediators.

#### 3.3.3. QUADAS-3 Appraisal of Susceptibility-Testing Studies

**Figure 4:**
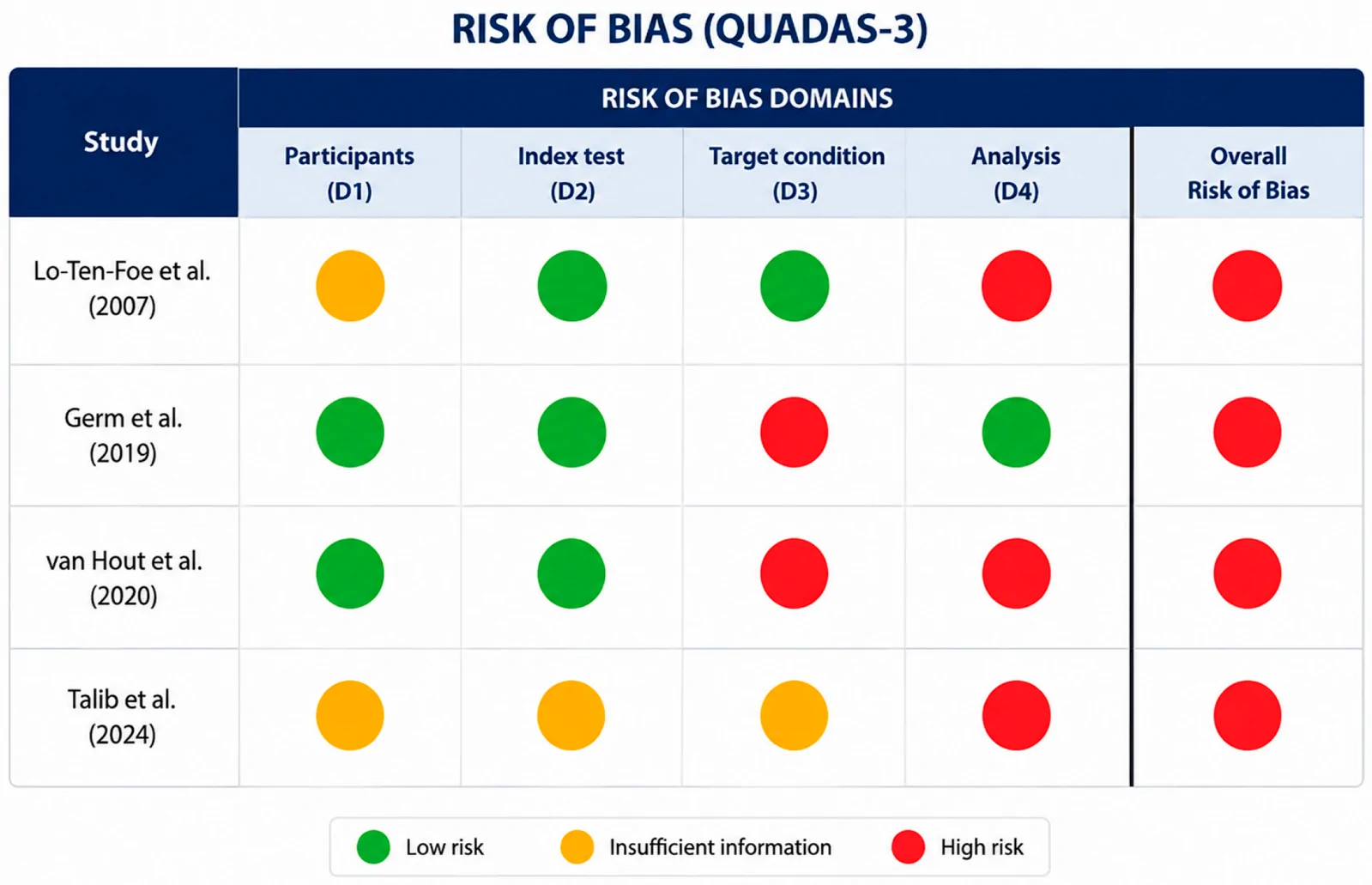
QUADAS-3 risk-of-bias assessment of the four diagnostic/AST comparisons. Full assessments are presented in Supplementary Table S2 [15,16,17,18].

All four QUADAS-3 AST comparisons were judged to be at high overall risk of bias (Figure 4), chiefly because of partial verification, the exclusion of difficult/discordant isolates, incomplete 2 × 2 data, or internal numerical inconsistencies. Applicability concerns were low in one and high in three. The most important problems were incomplete independent reference testing and lack of unambiguous contingency data. These limitations prevented a combined diagnostic accuracy estimate and justified study-level interpretation.

#### 3.3.4. MMAT 2018 Appraisal

**Figure 5:**
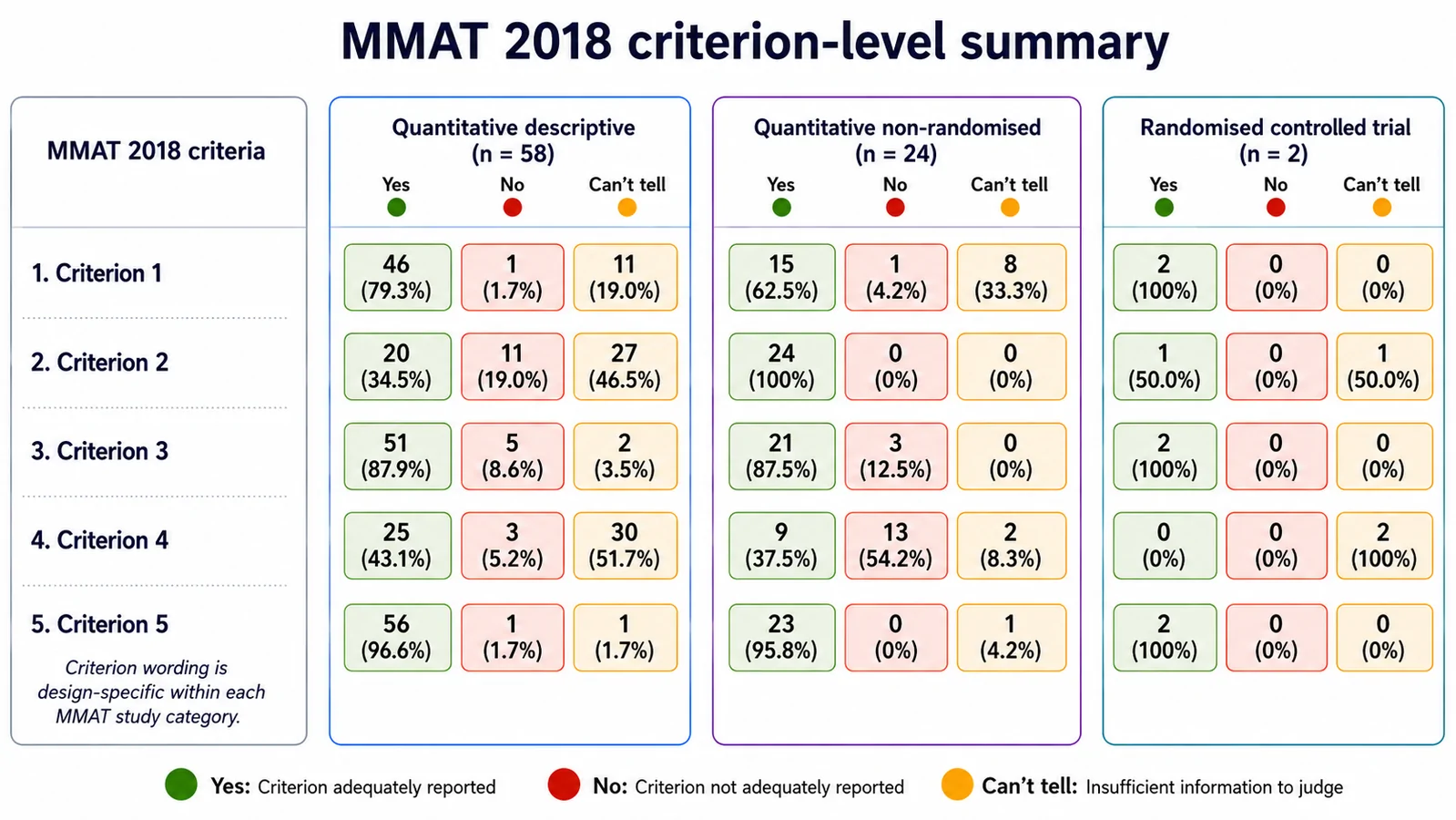
MMAT 2018 criterion-level appraisal by study design (Yes/No/Can’t tell). Full assessments are presented in Supplementary Table S2.

MMAT 2018 covered 58 quantitative descriptive, 24 quantitative non-randomised, and two randomised studies/components. Descriptive studies generally had appropriate measurement and analysis but often incompletely reported representativeness or non-response. Non-randomised studies usually measured exposures/outcomes adequately, but confounding was explicitly addressed in fewer than half. The two cluster-randomised studies generally met randomisation and intervention adherence criteria, with some reporting uncertainty (Figure 5). In the descriptive group, statistical analysis was judged adequate in 56/58 (96.6%) and measurement appropriate in 51/58 (87.9%), while representativeness and non-response were commonly unclear. Among 24 non-randomised assessments, exposure–outcome measurement was appropriate in all and outcome data were complete in 21/24 (87.5%), but confounding was explicitly accounted for in only 9/24 (37.5%).

### 3.4. Prevalence and Epidemiological Burden of Colistin Non-Susceptibility

Thirty-seven studies contributed prevalence/surveillance evidence [15,16,19,20,21,22,23,24,25,26,27,28,29,30,31,32,33,34,35,36,37,38,39,40,41,42,43,44,45,46,47,48,49,50,51,52,53]. Nineteen used reference or adequately documented BMD, 17 used non-reference or insufficiently documented methods, and one focused on heteroresistance. Because analytical unit, denominator, species, sampling frame, and AST differed, the findings were stratified rather than pooled.

Among broader ICU populations not selected for colistin resistance, 10 BMD studies yielded interpretable proportions (Table 2). Isolate-level estimates ranged from approximately 0.3% to 57.4%, while BMD-confirmed patient-level carriage/acquisition estimates ranged from approximately 1.2% to 9.4%. Variation was substantial across species and calendar periods, including very low resistance in some multicentre surveillance datasets and >50% for *K. pneumoniae* and *A. baumannii* bloodstream isolates in one study [23,24,36]. These ranges describe observed dispersion, not a pooled prevalence. Examples illustrate the scale of this variation. One multicentre Taiwanese dataset found colistin non-susceptibility of 0/121 *E. coli*, 3/137 (2.2%) *K. pneumoniae*, 16/51 (31.4%) *Enterobacter cloacae* complex, 6/128 (4.7%) *P. aeruginosa*, and 14/138 (10.1%) *A. baumannii* complex [36]. In contrast, a 2022–2024 adult-ICU bloodstream study reported 56.9% in *K. pneumoniae* and 57.4% in *A. baumannii* [23]. Another adult-ICU surveillance study showed marked period-to-period increases, particularly in *K. pneumoniae* and *A. baumannii* [24]. Patient-level BMD-confirmed estimates included 12/256 (4.7%) colistin-resistant carbapenemase-producing *Enterobacterales* carriers [38], 31/330 (9.4%) colonised patients [15], and 5/428 (1.2%) patients with acquired colistin resistance in a selective-decontamination cohort [50].

Five BMD studies used resistance-enriched denominators and reported approximately 2.1–63.0% resistance; four further BMD reports used selected, outbreak, overlapping, or incompletely defined denominators. Seventeen studies used non-reference or insufficiently documented AST and were only retained as method-cautious descriptive evidence. Heteroresistance was kept separate: one selected cohort reported 76/109 patients (69.7%) with the study-specific polymyxin-heteroresistant CRKP phenotype [20]. Overall, denominator definition and AST method were major determinants of the apparent burden. The complete epidemiological dataset is provided in Supplementary Table S3. Resistance-enriched estimates were intentionally not merged with the broader ICU estimates because selecting carbapenem-resistant, ESBL/carbapenemase-producing, multidrug-resistant, or otherwise resistant isolates changes the target population. Likewise, outbreak investigations and resistance-selected molecular collections were interpreted within their original sampling frames rather than treated as prevalence studies.

### 3.5. Clinical Outcomes and Factors Associated with Colistin Resistance

Forty-nine records contributed clinical outcomes, acquisition, or associated-factor evidence [32,37,42,45,47,49,50,52,54,55,56,57,58,59,60,61,62,63,64,65,66,67,68,69,70,71,72,73,74,75,76,77,78,79,80,81,82,83,84,85,86,87,88,89,90,91,92,93,94]. Eleven explicit exposure–outcome contrasts were suitable for ROBINS-E: ten compared outcomes in colistin-resistant versus susceptible infection/bacteraemia, and one evaluated prior colistin exposure and subsequent resistant colonisation. The remaining studies provided descriptive acquisition, treatment, outbreak, or clinical molecular evidence; no clinical effect was pooled. Full study-level data are presented in Supplementary Table S4.

The mortality findings were inconsistent. Crude mortality was substantially higher, with resistance in several cohorts (e.g., 70.0% vs. 48.6% [85], 100% vs. 50% [75], and 85% vs. 39% [76]). Other studies, however, showed little difference or the opposite direction (40.7% vs. 38.4% [87]; 46% vs. 56% [88]). Adjusted estimates were similarly divergent: HR 8.09 (95% CI 3.14–11.23) [63] and OR 8.56 (95% CI 1.98–39.03) [76] suggested increased mortality, whereas one study reported a cause-specific HR of 0.55 (95% CI 0.31–0.95) [88]. The first two were at high risk of bias and the latter had some concerns. A resistant-versus-susceptible CRAB bacteraemia study [74], an *Acinetobacter* infection series [32], a ventilator-associated pneumonia cohort [71], and a Gram-negative bloodstream infection cohort [81] also contributed unadjusted or incompletely adjusted contrasts. Differences in mortality horizon (ICU, in-hospital, 28-day, or 30-day), infection syndrome, organism, and susceptibility ascertainment further limited direct comparison.

For acquisition, 30/150 selected ICU patients were colonised with colistin-resistant *K. pneumoniae*. Prior colistin exposure was associated with colonisation (crude OR 6.98, 95% CI 2.0–24.3; adjusted OR 5.2, *p* < 0.001) [42]. However, ROBINS-E rated the result very high risk because of confounding by indication, prolonged-stay selection, and uncertain temporal alignment.

Broader clinical studies described resistance emergence after prolonged antimicrobial exposure, local transmission, or selective digestive decontamination, but the effects differed by setting [54,55,57,67,93,94]. Descriptive treatment series also reported substantial morbidity and mortality in established resistant infection without establishing an attributable resistance effect [32,37,61,66,89,90]. Recurrent associated factors included prior colistin or other broad-spectrum antibiotics, longer hospital/ICU exposure, invasive support, and outbreak/transmission context, but definitions and comparator structures were heterogeneous. Antoniadou et al. described 18 resistant *K. pneumoniae* isolates from 13 ICU patients after prolonged hospitalisation and colistin exposure, with six clones and evidence of horizontal transmission [54]. In a Dutch outbreak ICU, resistance emerged rapidly after the introduction of colistin-containing selective digestive decontamination [55]. In contrast, a low-endemicity multicentre setting reported low and similar respiratory acquisition rates under selective digestive/oropharyngeal decontamination and standard care [57]. Patient-level analyses reported associations with prior colistin or tigecycline, tracheostomy, pulmonary source, longer hospital stay, or unit-level aerosolised colistin use [67,93,94], although causal interpretation remained limited. Descriptive cohorts of resistant *A. baumannii*, resistant KPC-producing *K. pneumoniae*, and CRAB illustrated treatment complexity and high event rates, but were not designed to isolate the effect of colistin resistance [37,61,66,89,90].

Taken together, clinical evidence links colistin resistance to a high-risk ICU context but does not establish a uniform independent effect on mortality or acquisition. High/very high ROBINS-E risk in 10 of 11 evaluable contrasts, heterogeneous AST, and frequent absence of susceptible comparators support study-specific interpretation rather than causal or pooled estimates.

### 3.6. Susceptibility-Testing Methodology and Heteroresistance

Ten studies contributed AST-methodology or heteroresistance evidence [15,16,17,18,20,30,95,96,97,98]. Four method comparisons were appraised with QUADAS-3 and six descriptive/mechanistic heteroresistance components with MMAT. BMD was the preferred reference for conventional susceptibility, and population analysis profiling with MIC confirmation the reference framework for heteroresistance when available.

Method-dependent detection was substantial. In 28 *A. baumannii* isolates, repeated population analysis profiling identified 13 heteroresistant isolates, whereas the disc-based approach detected none of the 13 and yielded no false-positive heteroresistance among the 15 PAP-negative isolates [96]. VITEK 2 and other methods showed agreement limitations and incomplete verification [17]. In the VITEK 2 comparison, the principal analysis excluded *Stenotrophomonas maltophilia* that could not be interpreted and heteroresistant *E. cloacae* isolates, limiting generalisability [17].

Selective culture studies found higher recovery with SuperPolymyxin-based strategies, especially after enrichment: one study detected 17/45 resistant isolates by direct plating versus 45/45 after pre-enrichment [15], and another detected 4/9 by conventional culture versus 8/9 by SuperPolymyxin [16]. Because reference verification was incomplete or incorporated index test results, these are diagnostic yield comparisons rather than unbiased sensitivity estimates. Agar dilution versus BMD in 100 XDR isolates could not yield a unique 2 × 2 table because the publication contained internally inconsistent error/agreement counts [18]. Key comparisons are summarised in Table 3. In the 100-isolate agar-dilution study, BMD classified 11 isolates as resistant and 89 as susceptible, but published major/very-major-error counts, and the agreement values were mutually incompatible. The study was therefore only retained for narrative method interpretation [18]. The cohort contained two paediatric participants and was used solely for AST methodology, not adult prevalence or clinical outcomes.

Heteroresistance estimates varied sharply with organism and sampling frame. Reported examples included 76/109 (69.7%) selected CRKP patients [20], 108/288 (37.5%) randomly selected BMD-susceptible *K. pneumoniae* isolates from 13 European ICUs [95], 7/30 selected susceptible ICU isolates [98], and high proportions in selected susceptible *Acinetobacter* collections [30,97]. These values were not combined because the definitions, denominators, ward composition, and detection protocols differed. In the European multicentre collection, the 108 heteroresistant isolates arose from 288 randomly selected BMD-susceptible *K. pneumoniae* isolates [95]. Another study found seven heteroresistant *K. pneumoniae* among 30 selected BMD-susceptible ICU isolates, all missed by Rapid Polymyxin NP and negative for *mcr-1/2* [98]. In selected *A. baumannii* samples, 20/24 BMD-susceptible isolates were heteroresistant [97]. Akkan Kuzucu et al. similarly found resistant subpopulations in 26/93 BMD-susceptible *Acinetobacter* spp. isolates, but ICU-specific heteroresistance was not separable [30].

Overall, BMD remains the reference framework for conventional colistin classification, while heteroresistance requires dedicated methods. Selective media and enrichment can increase recovery, but diagnostic yield should not be labelled sensitivity/specificity without independent verification of eligible positives and negatives. Complete study-level AST and heteroresistance evidence is provided in Supplementary Table S6.

### 3.7. Molecular Determinants and Epidemiological Patterns

Fifty-four studies contributed molecular/mechanistic evidence [26,30,47,49,50,52,53,54,60,61,64,65,67,68,70,92,93,96,99,100,101,102,103,104,105,106,107,108,109,110,111,112,113,114,115,116,117,118,119,120,121,122,123,124,125,126,127,128,129,130,131,132,133,134]. Most used selected resistant isolates, sequencing-enriched subsets, or outbreak collections; determinant frequencies therefore describe tested molecular samples, not ICU population prevalence.

Mobile determinants included *mcr-1*/*mcr-1.1*, *mcr-8*, and *mcr-9* in ICU-linked *Enterobacterales* and, rarely, *A. baumannii* [101,107,119,124,130,131]. Gene carriage did not consistently predict resistance: phenotypically susceptible *mcr*-positive isolates and both resistant and susceptible *mcr-9* backgrounds were reported [124,130]. In a selected resistant *K. pneumoniae* series, *mcr-1* was present in 19/22 isolates and the remaining three had *mgrB* disruption [101]. Other reports identified susceptible *mcr-1.1* carriers [130], plasmid-borne *mcr-1.1* in *A. baumannii* [119], and ICU-linked *mcr-8* or *mcr-9* [107,124,131].

Chromosomal resistance was prominent in *K. pneumoniae*, especially *mgrB* disruption/truncation, with additional Pmr/Bas pathway alterations [50,64,99,117,121,126,133]. In *A. baumannii*, PmrAB/PmrC signalling, lpx pathways, and lipid-A remodelling were repeatedly implicated [61,68,112,118,134]. Individual variants were heterogeneous, supporting multiple routes to a common membrane-modification phenotype rather than one universal mutation. Same-isolate data provided the clearest evidence: all 10 resistant isolates in one KPC-producing *K. pneumoniae* genomic series had an IS5-like *mgrB* interruption absent from six susceptible isolates [99], and *mgrB* disruption predominated in an ST258-resistant series [64]. Further ICU studies documented *mgrB* or Pmr/Bas alterations in resistant *Enterobacterales* [117,121,126,133]. In *A. baumannii*, studies linked resistance to *pmrB*/*pmrC* dysregulation, *lpx* changes, or phosphoethanolamine modification of lipid A. These studies also showed that some candidate substitutions occur in susceptible backgrounds [61,68,112,118,134].

Genomic epidemiology documented both clonal dissemination and independent evolution [47,52,60,99,109,110]. Longitudinal paired-isolate studies captured within-host susceptible-to-resistant transitions through *mgrB*, *basRS*/*pmrAB*, or heteroresistant subpopulations [50,61,96,129]. Candidate resistance-associated variants were also observed in susceptible backgrounds [53,103,105], reinforcing the need for same-isolate genotype–phenotype interpretation. Outbreak and typing studies ranged from single-clade dissemination to multiclonal resistant populations [47,52,60,99,109,110]. Within-host analyses were especially informative: paired susceptible/resistant *A. baumannii* isolates supported de novo evolution [61]; a five-patient *Enterobacterales* series identified several evolutionary routes including *mcr-1.1* acquisition and *basRS/pmrAB* changes [50], and a later *K. pneumoniae* isolate developed IS5-mediated *mgrB* disruption [129]. Conversely, candidate resistance-related variants in *P. aeruginosa* and *A. baumannii* and *mcr-1* carriage in mixed phenotypic backgrounds illustrated recurrent genotype–phenotype discordance [53,103,105].

Overall, colistin resistance reflects a plural molecular model: transferable *mcr* determinants coexist with chromosomal regulatory/lipid-A pathways, clonal spread, and within-host evolution. Molecular testing can clarify mechanism and transmission but should complement, not replace, reference phenotypic susceptibility testing. Complete molecular extraction is provided in Supplementary Table S5.

## 4. Discussion

### 4.1. Principal Findings

This review integrates epidemiological, clinical, laboratory, and molecular evidence on colistin resistance in critically ill adults. Its central finding is that resistance cannot be represented by a single ICU rate or effect estimate: apparent burden and clinical meaning depend on denominator, organism, sampling frame, AST method, and study design. In contrast to the previous prevalence-focused meta-analysis [8], the present synthesis separates methodologically distinct domains and interprets them together. This distinction is clinically important because two studies can report substantially different proportions while both being internally valid if they sampled different organisms, resistance-enriched populations, or units of analysis. The review therefore treats heterogeneity as a finding to be explained rather than a nuisance to be eliminated through pooling.

Even among broader BMD-tested ICU populations, isolate-level non-susceptibility ranged from approximately 0.3% to 57.4% and patient-level carriage/acquisition estimates from approximately 1.2% to 9.4%. Clinical associations with mortality were inconsistent and mostly at high or very high risk of bias. Laboratory studies confirmed method-dependent detection and the special challenge of heteroresistance, while molecular studies identified multiple transferable and chromosomal pathways with meaningful genotype–phenotype discordance.

### 4.2. Epidemiological Burden and the Problem of a Single Prevalence Estimate

The epidemiological range primarily reflects heterogeneity rather than a common prevalence parameter. Geography is important [8], but so are analytical unit, species, resistance enrichment, colonisation versus infection, study period, and AST. This accords with the broader global surveillance context of substantial and regionally variable antimicrobial resistance [135]. The decision not to calculate a single pooled prevalence is therefore methodological rather than merely statistical: pooling would obscure whether observed differences arose from biology, selection of the denominator, or measurement.

Resistance-enriched denominators answer a different question from unselected ICU surveillance. The approximately 2.1–63.0% proportions observed in carbapenem-/multidrug-resistant subgroups quantify loss of colistin activity within already difficult-to-treat populations, not overall ICU prevalence. Patient- and isolate-level estimates are likewise not interchangeable because isolate counts depend on sampling intensity and specimen distribution.

Accordingly, surveillance should report explicit patient- or isolate-level denominators, organism, specimen/infection context, calendar period, and AST method. Species- and setting-specific estimates are more interpretable than a universal ICU prevalence.

### 4.3. Clinical Outcomes and Associated Factors

The available clinical literature cannot determine whether colistin resistance independently worsens prognosis or mainly marks patients with greater baseline risk. Both crude and adjusted mortality estimates varied widely, and none of the 11 ROBINS-E contrasts was at low risk of bias. Large effect estimates from small or selected cohorts may be clinically concerning. However, their magnitude should not be transferred to other ICU populations without accounting for illness severity, infection source, co-resistance, availability and timing of active therapy, and competing risks.

Residual confounding, prolonged-stay or survivor selection, uncertain time zero, and adjustment for post-exposure treatment/source control were recurrent problems. In addition, survival requirements can introduce time-dependent or immortal-time bias in acute infection studies [136]. Prior colistin exposure is similarly difficult to interpret because confounding by indication links treatment to illness severity, prior antimicrobial burden, and resistant organism exposure.

Thus, current evidence supports an association with a high-risk clinical context rather than a uniform causal mortality effect. Future studies should predefine time zero, exposure, comparator, and outcome; measure baseline severity and co-resistance; and distinguish baseline confounders from post-exposure mediators.

### 4.4. Susceptibility Testing and Heteroresistance

AST methodology is integral to epidemiological and clinical interpretation. Several routine approaches are not equivalent to reference BMD; therefore, between-study variation can reflect measurement as well as biology. All four QUADAS-3 comparisons were at high risk of bias, chiefly because of partial verification, incomplete 2 × 2 reporting, or the exclusion of discordant isolates. The method comparison evidence also illustrates why routine laboratory categories should not be assumed equivalent across studies. A method that under-detects resistance, for example, can lower an epidemiological numerator and simultaneously misclassify the exposure used in a clinical outcome analysis.

Heteroresistance further complicates routine classification. Selected ICU studies reported widely varying proportions, and a separate meta-analysis in *K. pneumoniae* also found a high pooled proportion with extreme between-study heterogeneity [137]. Longitudinal observations suggest that resistant subpopulations may be selected during polymyxin exposure, but present data do not justify a universal ICU heteroresistance rate.

Method comparison studies should independently apply index and reference tests to eligible samples, verify negatives as well as positives, report complete contingency data, and use harmonised heteroresistance definitions and reference procedures.

### 4.5. Molecular Mechanisms, Transmission, and Genotype–Phenotype Discordance

Molecular evidence likewise does not support a single mechanism. *mgrB* disruption and Pmr/Bas pathways were frequent in resistant *Enterobacterales*, whereas Pmr/lpx/lipid-A pathways were prominent in *A. baumannii*. Mobile *mcr* genes add horizontal-transfer potential; their wider circulation across human, animal, and environmental reservoirs is relevant to the clinical resistance reservoir [138]. Similar phenotypes may therefore arise by horizontal acquisition, clonal transmission, or independent chromosomal evolution. This distinction matters for infection prevention and for the interpretation of molecular surveillance.

However, *mcr* carriage and candidate chromosomal variants were sometimes present in susceptible isolates. Genomic investigations also documented both clonal spread and multiple independent lineages, while longitudinal paired isolates captured within-host evolution. Infection control conclusions therefore require typing evidence rather than inference from resistance phenotype alone.

The most informative molecular studies combine reference phenotyping with longitudinal genomics. Phenotypic AST establishes clinical susceptibility, whereas sequencing can identify mechanism, relatedness, transmission, and within-host evolution; the two are complementary.

### 4.6. Integration of the Four Evidence Domains

The four evidence domains are interdependent but not interchangeable. AST determines the phenotype used in prevalence and clinical analyses. Molecular data can explain transmission or evolution, but do not provide population prevalence when based on selected isolates. Epidemiological studies without molecular typing, in turn, cannot distinguish clonal expansion from independent emergence.

This layered framework extends prior prevalence-focused work [8] by showing why denominator and laboratory methods must accompany any reported rate and why clinical, diagnostic, heteroresistance, and molecular findings require their own analytical rules.

### 4.7. Strengths and Limitations

Strengths include a four-database search up to 3 August 2026 without date or language restrictions, 120 studies across four prespecified domains, and separation of patient- and isolate-level denominators and resistance-enriched samples. Additional strengths include the prioritisation of BMD for epidemiological interpretation and design-specific appraisal with JBI, ROBINS-E, QUADAS-3, and MMAT. Molecular determinants were only linked to phenotype when evaluated in the same isolates. The domain-based structure also prevented study-specific molecular frequencies, diagnostic yields, and selected resistant cohorts from being mistaken for population-level estimates.

Limitations include the absence of prospective registration, 19 unretrieved reports, and substantial heterogeneity in ICU populations, organisms, colonisation/infection status, AST methods, breakpoints, periods, and geography. This heterogeneity precluded the meaningful pooling of prevalence and clinical effects.

Methodological limitations were also substantial: most prevalence studies had moderate/high concern, 10/11 ROBINS-E contrasts were high/very high risk, and all four QUADAS-3 comparisons were high risk. Molecular studies often used selected resistant or outbreak isolates, and heteroresistance definitions and protocols varied. Consequently, neither molecular determinant frequencies nor heteroresistance proportions can be generalised to unselected ICU populations.

### 4.8. Implications for Research and Clinical Practice

Future epidemiological studies should use explicit patient- or isolate-level denominators, separate colonisation from infection, report species-specific estimates, and use reference or validated AST; multicentre designs would improve generalisability. Clinical cohorts should use prospective causal designs with clearly aligned time zero, exposure, comparator, outcome, baseline severity, and antimicrobial history.

AST studies should apply independent reference testing to index-positive and index-negative samples and report complete 2 × 2 data. Heteroresistance research needs harmonised definitions and prospective evaluation of whether it predicts conventional resistance or clinical outcomes. Molecular surveillance should integrate BMD with the longitudinal sequencing of paired susceptible/resistant isolates.

Clinically, colistin susceptibility should be established with reference or adequately validated BMD rather than unreliable gradient- or disc-diffusion methods [7]. Heteroresistance should be considered when clinical response conflicts with an apparently susceptible result. Consistent with current guidance, newer active agents are increasingly preferred when available and appropriate [5]. Overall, progress depends on methodologically harmonised studies that integrate reliable phenotyping, clinically meaningful denominators, robust outcome assessment, and molecular characterisation.

## 5. Conclusions

Colistin resistance in critically ill adults is highly heterogeneous across epidemiological, clinical, laboratory, and molecular domains. Reported non-susceptibility varies with species, denominator, resistance enrichment, setting, calendar period, and AST method, making a single ICU prevalence estimate misleading.

Clinical studies do not show a uniform independent mortality effect and are frequently at high risk of bias. BMD remains the reference framework for phenotypic classification; heteroresistance requires dedicated methods; and resistance can arise through mobile *mcr* genes, *mgrB* disruption, Pmr/Pho/Bas and lipid-A pathways, clonal spread, or within-host evolution. Because genotype and phenotype can be discordant, integrated phenotypic and molecular characterisation is preferable. More standardised populations, AST methods, causal analyses, and longitudinal genomic designs are needed to improve comparability and clinical interpretation.

## Figures and Tables

**Table 1:** Summary characteristics of the 120 studies included in the systematic review.

Characteristic	Category	Studies
Publication period	2007–2014	12 (10.0%)
	2015–2019	33 (27.5%)
	2020–2026	75 (62.5%)
Geographical setting	Türkiye	26 (21.7%)
	India	13 (10.8%)
	Iran	10 (8.3%)
	Greece	9 (7.5%)
	The Netherlands	8 (6.7%)
	Brazil	7 (5.8%)
	Egypt	7 (5.8%)
	China	6 (5.0%)
	Other geographic entries	34 (28.3%)
Study design	Molecular/isolate-series epidemiology	38 (31.7%)
	Prevalence/surveillance	22 (18.3%)
	Other cohort, case–control, cross-sectional, ecological, outbreak, AST-comparison, and trial-derived designs	60 (50.0%)
Frequently represented organism *	*Klebsiella pneumoniae*	60
	*Acinetobacter baumannii*	45
	*Pseudomonas aeruginosa*	19
	*Escherichia coli*	14
	*Enterobacter cloacae* complex	5
Evidence domain **	Epidemiological prevalence/surveillance	37
	Clinical outcomes/acquisition/associated factors	49
	AST methodology/heteroresistance	10
	Molecular/mechanistic	54

* Organism categories are study-level mentions and are not mutually exclusive; a single study may report more than one organism. ** Evidence-domain categories are not mutually exclusive. The 120 studies generated 150 study-domain contributions: 95 studies contributed to one domain, 20 to two domains, and 5 to three.

**Table 2:** Higher-confidence epidemiological estimates of colistin non-susceptibility based on BMD in broader ICU populations.

Study	Population/Denominator	Key Colistin Finding	AST Method	Interpretation
Özkılıç et al. (2025) [23]	ICU Gram-negative bloodstream isolates; species-specific denominators	*Kp* 95/167 (56.9%); *Ab* 35/61 (57.4%); *Pa* 3/23 (13.0%)	Sensititre BMD; EUCAST	Broader ICU isolate-level BMD evidence (JBI: Moderate)
Özçelik et al. (2026) [24]	Adult ICU healthcare-associated Gram-negative isolates across three periods	*Kp* 2.8%, 34.0%, 32.1%; *Ab* 1.6%, 8.0%, 23.9%; *Pa* 1.3%, 1.9%, 15.2%	BMD; EUCAST	Temporal and species-specific BMD evidence (JBI: Moderate)
Ibrahim et al. (2021) [26]	100 Gram-negative ICU isolates	14/100 (14.0%)	ComASP BMD	Broader ICU isolate-level BMD evidence (JBI: Moderate)
Akkan Kuzucu et al. (2022) [30]	76 ICU *Acinetobacter* spp. isolates from a biobank	10/76 (13.2%)	BMD; EUCAST	ICU-specific BMD estimate; biobank frame incompletely described (JBI: Moderate)
Liao et al. (2019) [35]	300 consecutive non-duplicate ICU isolates: 100 *E. coli*, 100 *K. pneumoniae*, 100 *P. aeruginosa*	1/300 (0.3%) overall; *Kp* 1/100, *Ec* 0/100, *Pa* 0/100	Sensititre BMD; CLSI	Broader multicentre ICU isolate-level BMD evidence (JBI: Moderate)
Lai et al. (2019) [36]	758 non-duplicate Gram-negative ICU isolates; acquired-resistance species analysed separately	*Ec 0/121; Kp 3/137 (2.2%); Ecl 16/51 (31.4%); Pa 6/128 (4.7%); Ab* complex 14/138 (10.1%)	Reference BMD	Species-specific multicentre ICU BMD evidence (JBI: Moderate)
Unlu et al. (2021) [41]	83 consecutive non-duplicate ICU *K. pneumoniae* isolates	15/83 (18.1%)	BMD; EUCAST	Broader ICU *K. pneumoniae* BMD evidence (JBI: Moderate)
Dos Santos et al. (2024) [38]	256 consecutive ICU patients in a multicentre point-prevalence study	COLr-CPE carriage 12/256 patients (4.7%)	UMIC BMD; EUCAST	Patient-level ICU carriage estimate (JBI: Low)
Germ et al. (2019) [15]	330 ICU patients undergoing surveillance cultures	31/330 colonised (9.4%)	BMD confirmation	Patient-level BMD-confirmed carriage estimate (JBI: Low)
Janssen et al. (2020) [50]	428 ICU patients; 286 isolates after excluding intrinsically resistant species	5/428 patients (1.2% carriage); 13/286 isolates (4.5%)	Sensititre with BMD confirmation; EUCAST	Distinct patient- and isolate-level estimates; overlapping companion report handled separately (JBI: Low)

Note: JBI categories are review-specific operational classifications, not official numerical scores. Supplementary Table S3 contains all prevalence/surveillance studies, including non-reference AST, resistance-enriched, heteroresistance, outbreak/selected, and overlapping datasets. Abbreviations: *Kp*, *K. pneumoniae*; *Ab*, *A. baumannii*; *Pa*, *P. aeruginosa*; *Ec*, *E. coli*; *Ecl*, *E. cloacae*.

**Table 3:** Key susceptibility-testing and screening method comparisons evaluated with QUADAS-3.

Study	Population/Unit	Index Method	Reference/Confirmation	Key Finding	QUADAS-3	Role in Synthesis
Lo-Ten-Foe et al., 2007 [17]	102 Gram-negative ICU-linked isolates	VITEK 2 and other AST methods	BMD (CLSI)	MIC agreement reported, but no unique complete 2 × 2; heteroresistant *E. cloacae* and *S. maltophilia* excluded from main analysis	High; applicability high	Narrative agreement and method limitations only
Germ et al., 2019 [15]	739 surveillance samples from 330 ICU patients	Direct plating vs. overnight pre-enrichment on SuperPolymyxin	BMD confirmation of recovered isolates; composite positivity standard	Direct plating recovered 17/45 resistant isolates (37.8%), whereas pre-enrichment recovered 45/45 (100%); because the reference process was not independent for all samples, these data are interpreted as diagnostic yield rather than formal sensitivity.	High; applicability low	Supports higher yield after pre-enrichment; not absolute accuracy
van Hout et al., 2020 [16]	1105 consecutive rectal swabs from 428 ICU patients	Conventional culture vs. SuperPolymyxin	BMD confirmation among isolates recovered by either method	4/9 vs. 8/9 resistant isolates detected; combined methods 9/9	High; applicability high for formal DTA	Narrative diagnostic yield evidence; specificity not validly estimable
Talib et al., 2024 [18]	100 XDR Gram-negative isolates; adult ICU cohort + 2 paediatric	Agar dilution	BMD (CLSI)	BMD: 11 resistant/89 susceptible; internally inconsistent major error and agreement data prevent a unique 2 × 2 reconstruction	High; applicability high	Narrative AST-method evidence only; excluded from adult prevalence and clinical-outcome synthesis

QUADAS-3 judgements refer to the specific comparison and were not converted to a numerical score. Supplementary Table S6 contains all 10 studies: four QUADAS-3 method comparisons and six MMAT-appraised heteroresistance studies.

## Data Availability

No new datasets were generated during this study. All data analysed in this systematic review were derived from previously published studies. The extracted data supporting the findings are provided in the manuscript and Supplementary Tables S1–S6.

## Supplementary Materials

The following supporting information can be downloaded at https://www.mdpi.com/article/10.3390/microorganisms14091908/s1: Table S1: Complete database-specific search strategies; Table S2: Methodological appraisal of included studies; Table S3: Complete study-level prevalence and surveillance evidence (37 studies); Table S4: Clinical outcomes, acquisition/risk-factor findings, treatment, and outbreak-context evidence in the clinical domain (49 records); Table S5: Study-level molecular determinants, molecular epidemiology, and genotype–phenotype interpretation (54 studies); Table S6: Susceptibility-testing methodology and heteroresistance evidence (10 studies); File S1: PRISMA 2020 checklist.

## Abbreviations

The following abbreviations are used in this manuscript:

AMR	antimicrobial resistance
AP-PCR	arbitrarily primed polymerase chain reaction
AST	antimicrobial susceptibility testing
BMD	broth microdilution
BSI	bloodstream infection
CI	confidence interval
CLSI	Clinical and Laboratory Standards Institute
COLr-CPE	colistin-resistant carbapenemase-producing *Enterobacterales*
CRAB	carbapenem-resistant *Acinetobacter baumannii*
CR-GNB	carbapenem-resistant Gram-negative bacteria
CRKP	carbapenem-resistant *Klebsiella pneumoniae*
DTA	diagnostic test accuracy
ESBL	extended-spectrum beta-lactamase
EUCAST	European Committee on Antimicrobial Susceptibility Testing
HR	hazard ratio
ICU	intensive care unit
JBI	Joanna Briggs Institute
KPC	*Klebsiella pneumoniae* carbapenemase
MIC	minimum inhibitory concentration
MLST	multilocus sequence typing
MMAT	Mixed Methods Appraisal Tool
OR	odds ratio
PAP	population analysis profiling
PFGE	pulsed-field gel electrophoresis
PRISMA	Preferred Reporting Items for Systematic Reviews and Meta-Analyses
QUADAS-3	Quality Assessment of Diagnostic Accuracy Studies, version 3
ROBINS-E	Risk Of Bias In Non-randomised Studies—of Exposure
VAP	ventilator-associated pneumonia
WGS	whole-genome sequencing
XDR	extensively drug-resistant
