# Supplementary material for: Colistin Resistance in Critically Ill Adults: A Systematic Review of Epidemiological, Clinical, Laboratory, and Molecular Evidence
Source: Microorganisms. 2026 Aug 28;14(9):1908. doi: 10.3390/microorganisms14091908 (PMC13609447; doi:10.3390/microorganisms14091908)
Supplement: Supplementary file 1 [file microorganisms-14-01908-s001.zip › Supplementary_Table_S1_Search_Strategies.pdf]

Supplementary Table S1. Complete database-specific search strategies

The database-specific search strategies below are reproduced as documented in the final search records supplied by the authors.

Database	Interface / field syntax
**MEDLINE via PubMed**	MeSH + title/abstract [tiab]
**Cochrane Library**	MeSH descriptors + ti,ab,kw; stepwise #1-#12
**Scopus**	TITLE-ABS-KEY
**Web of Science Core Collection**	TS= topic search

MEDLINE via PubMed

```
(
 "Colistin"[Mesh]
 OR colistin[tiab]
 OR "polymyxin E"[tiab]
)
AND
(
 "Intensive Care Units"[Mesh]
 OR "Critical Illness"[Mesh]
 OR "Critical Care"[Mesh]
 OR ICU[tiab]
 OR ICUs[tiab]
 OR "intensive care"[tiab]
 OR "critical care"[tiab]
 OR "critically ill"[tiab]
 OR "mechanically ventilated"[tiab]
 OR "mechanical ventilation"[tiab]
)
AND
(
 "Drug Resistance, Bacterial"[Mesh]
 OR "Drug Resistance, Microbial"[Mesh]
 OR "colistin resistance"[tiab]
 OR "colistin resistant"[tiab]
 OR "colistin-resistant"[tiab]
 OR "colistin nonsusceptib*"[tiab]
 OR "colistin non-susceptib*"[tiab]
 OR heteroresistan*[tiab]
 OR "mcr-1"[tiab]
 OR "mcr-2"[tiab]
 OR "mcr-3"[tiab]
 OR "mcr-4"[tiab]
 OR "mcr-5"[tiab]
 OR "mcr-6"[tiab]
 OR "mcr-7"[tiab]
 OR "mcr-8"[tiab]
 OR "mcr-9"[tiab]
 OR "mcr-10"[tiab]
 OR "mcr gene*"[tiab]
 OR mgrB[tiab]
 OR phoP[tiab]
 OR phoQ[tiab]
 OR phoPQ[tiab]
 OR pmrA[tiab]
 OR pmrB[tiab]
 OR pmrAB[tiab]
 OR crrA[tiab]
 OR crrB[tiab]
 OR lpxA[tiab]
 OR lpxC[tiab]
 OR lpxD[tiab]
)
```

Cochrane Library

Step	Exact search syntax
**#1**	MeSH descriptor: [Colistin] explode all trees
**#2**	(colistin OR "polymyxin E"):ti,ab,kw
**#3**	#1 OR #2
**#4**	MeSH descriptor: [Critical Illness] explode all trees
**#5**	MeSH descriptor: [Intensive Care Units] explode all trees
**#6**	MeSH descriptor: [Critical Care] explode all trees
**#7**	(ICU OR ICUs OR "intensive care" OR "intensive care unit" OR "intensive care units" OR "critical care" OR "critical care unit" OR "critical care units" OR "critically ill" OR "mechanically ventilated" OR "mechanical ventilation"):ti,ab,kw
**#8**	#4 OR #5 OR #6 OR #7
**#9**	MeSH descriptor: [Drug Resistance, Bacterial] explode all trees
**#10**	("colistin resistance" OR "colistin resistant" OR "colistin-resistant" OR "colistin non-susceptible" OR "colistin non-susceptibility" OR "colistin nonsusceptible" OR "colistin nonsusceptibility" OR heteroresistance OR heteroresistant OR "mcr-1" OR "mcr-2" OR "mcr-3" OR "mcr-4" OR "mcr-5" OR "mcr-6" OR "mcr-7" OR "mcr-8" OR "mcr-9" OR "mcr-10" OR "mcr gene" OR "mcr genes" OR mgrB OR phoPQ OR pmrAB):ti,ab,kw
**#11**	#9 OR #10
**#12**	#3 AND #8 AND #11

Scopus

TITLE-ABS-KEY (colistin OR "polymyxin E") AND TITLE-ABS-KEY (ICU OR ICUs OR "intensive care" OR "intensive care unit*" OR "critical care" OR "critical care unit*" OR "critically ill" OR "mechanically ventilated" OR "mechanical ventilation") AND TITLE-ABS-KEY ("colistin resistan*" OR "colistin non-susceptib*" OR "colistin nonsusceptib*" OR heteroresistan* OR "mcr-1" OR "mcr-2" OR "mcr-3" OR "mcr-4" OR "mcr-5" OR "mcr-6" OR "mcr-7" OR "mcr-8" OR "mcr-9" OR "mcr-10" OR "mcr gene*" OR mgrB OR phoPQ OR pmrAB)

Web of Science Core Collection

```
TS=(colistin OR "polymyxin E")
AND TS=(
 ICU
 OR ICUs
 OR "intensive care"
 OR "intensive care unit*"
 OR "critical care"
 OR "critical care unit*"
 OR "critically ill"
 OR "mechanically ventilated"
 OR "mechanical ventilation"
)
AND TS=(
 "colistin resistan*"
 OR "colistin non-susceptib*"
 OR "colistin nonsusceptib*"
 OR heteroresistan*
 OR "mcr-1"
 OR "mcr-2"
 OR "mcr-3"
 OR "mcr-4"
 OR "mcr-5"
 OR "mcr-6"
 OR "mcr-7"
 OR "mcr-8"
 OR "mcr-9"
 OR "mcr-10"
 OR "mcr gene*"
 OR mgrB
 OR phoPQ
 OR pmrAB
)
```
